# Long-term effects of the COVID-19 lockdown on weight status, eating habits, and lifestyle changes related to school-aged children in Bandar Abbas, Iran

**DOI:** 10.1186/s12889-024-19509-3

**Published:** 2024-07-24

**Authors:** Behnaz Khamesan, Niloufar Khatibzade-Nasari, Shahram Zare, Narges Rostami-Gooran, Roya Baghestani-Koozehgar

**Affiliations:** 1 Department of medicine, Qeshm branch, Islamic Azad University, Qeshm, Iran; 2 Community Medicine Department, School of Medicine, Hormozgan University of Medical Sciences, Bandar Abbas, Iran; 3 Community-Based Participatory Research Center, Tehran University of Medical Science, Tehran, Iran

**Keywords:** COVID-19, Lockdown, Children, School, Obesity, Overweight, Weight status, Lifestyle

## Abstract

**Background:**

Despite the end of the COVID-19 lockdown and reopening of schools, the long-term effects of quarantine on the weight status, eating habits, and lifestyle of children and adolescents remain unknown. This study aimed to determine the long-term effects of the lockdown on the weight status, eating habits, and lifestyle changes of children and adolescents in Iran.

**Method:**

This descriptive cross-sectional study was conducted from April to May 2022. The target population comprised one hundred students aged between 10 and 16 years old. Our study obtained students’ weight and height data from records maintained by school principals or physical education instructors before and after the quarantine period. The BMI z-score (zBMI) was calculated for each time point. The researchers also provided a questionnaire to collect the students’ demographic and lifestyle status changes during school closures.

**Results:**

We found that the zBMI increased significantly from − 0.02 ± 1.64 to 0.36 ± 1.12, and the number of individuals with overweight and obesity increased by 3% during quarantine (*P* ≤ 0.05). These changes were more pronounced in males and students aged 14–16 years old. We also found that eating habits, sleeping time, sleeping patterns, screen time (time spent on social media per day), and physical activity had significant negative changes during quarantine, and a significant increase in zBMI was observed among students who experienced negative eating behaviors, altered sleeping patterns, and decreased physical activity during school closures.

**Conclusion:**

As prolonged school closures due to the COVID-19 lockdown aggravated students’ health and lifestyle status, our findings can aid in proper planning to establish an appropriate framework for the diet, physical activity, and sleeping quality of students during extended school closures.

**Supplementary Information:**

The online version contains supplementary material available at 10.1186/s12889-024-19509-3.

## Background

The outbreak of coronavirus disease, resulting from severe acute respiratory syndrome coronavirus 2 (SARS-CoV-2), was first reported in Wuhan, China, in 2019, and the World Health Organization (WHO) reported the disease as a global pandemic in March 2020 [1].

Children significantly faced the consequences of this epidemic as a particular population. The possibility of children confronting the severe form of this disease is low, and it has been found that the transmission rate of the disease is not a significant concern in schools and childcare centers [2]. While almost all the world’s countries have been affected by the disease, some were more affected by the consequences of it than others. Iran was one of the countries that was widely affected by this pandemic at the early stages. Several surveys were taken in the country to prevent the spread of the virus, which mainly included the quarantine of cities and the closure of schools, religious places, judiciary systems, and offices to temporarily reduce the incidence of the virus [3]. However, unlike many businesses, schools were closed from the beginning of the first peak of this pandemic, leading to numerous problems, such as disruption in learning, changes in lifestyle, and increased stress and anxiety among children [2]. Moreover, as schools play an effective role in assuaging youth’s obesity risk, their closure affects the activity patterns and weight status of students by the time of lockdown [3].

Recent studies have revealed destructive changes in children’s eating habits, screen exposure, sleeping status, psychological responses, and activity behaviors during the lockdown and how these changes aggravated their health status [4–7]. Despite the end of the lockdown and reopening of schools, the long-term effects of quarantine on the lifestyle and weight status of children and adolescents remain unclear. Additionally, there is a possibility that these negative changes could become more prominent during extended school holidays such as summer breaks. Some studies mentioned that the same changes observed throughout the pandemic lockdown were also observed during summer breaks when schools were closed [8]. Therefore, it is necessary to identify factors affecting weight and lifestyle changes and try to reduce them by providing appropriate solutions.

While previous studies investigated the short-term impacts of the lockdown on the weight status and lifestyle of children and adolescents and relied on self-reported weight changes [1, 3, 9], our study aimed to assess the long-term impacts of the lockdown on weight status changes, utilizing weight and height data from school records. Additionally, we analyzed the risks associated with weight changes, including variations in eating habits, sleeping status, physical activity, and the use of multimedia devices.

## Method

### Design and rationale

The study was conducted from April to May 2022 in primary and junior middle schools in District 1 of Bandar Abbas City, Iran. The target population consisted of one hundred students. We used a multi-stage sampling technique to select schools, classes, and students from each stratum by picking 10 classes using cluster sampling and then randomly selecting 10 students from each class. The inclusion criteria consisted of students aged 10 to 16 years old along with the possibility of accessing their recorded height and weight that was done by the school principal or the physical education instructor right before and after the school closure at two time points, January-February 2020 and April-May 2022, as well as the consent from parents and assent from students to participate in the study. The Ethics Committee of Qeshm International University of Medical Sciences approved the study (IR.IAU.BA.REC.1402.010), and all information was collected using the included questionnaires and recorded student files.

After confirming the availability of information on the student’s weight and height before and after the lockdown, the cases were included in the study. Furthermore, if parental and student consent was obtained, the designed questionnaire was provided to them. According to the method of accessing the students, the relevant questionnaire was either completed in person or, in the case of lack of access, through phone follow-up by the students or their parents.

### Survey questionnaire

The questionnaire, which was designed to assess changes in students’ activities and priorities during quarantine, consisted of six sections. The first section covered demographic information, including the student’s sex, date of birth, parents’ occupational status, type of residence, and number of children in the family. The second part delved into students’ dietary habits, with questions about overeating under stress, food responsiveness, enjoyment of food, and appetite, evaluated based on a 5-point Likert scale (from never to always and strongly disagree to strongly agree). The third part analyzed the number of snacks consumed between main meals during the day (once a day, twice a day, 3 times a day, and 4 or more than 4 times a day) and the week (less than once a week, 1–3 times in a week, 4–6 times in a week, and every day). The corresponding questions were based on a recent study conducted in France [10]. The fourth part assessed the participants’ sleeping status, including their sleeping patterns, duration, and quality. The questions about sleeping quality (frequent waking periods during sleep, Nightmares during sleep, and difficulty falling asleep) were designed and asked in detail based on an Italian survey [11]. In the fifth part of the questionnaire, students were asked about their screen time before and during the lockdown, using a 4-point scale ranging from 0 to 1 h to more than 3 h per day. Lastly, the amount of physical activity and exercise intensity of the students before and during the lockdown was assessed and categorized. These categories included sedentary activities (almost all sitting-based activities), light-intensity activities (such as domestic or occupational tasks like washing dishes, hanging laundry, ironing, cooking, eating, and working at a computer desk), moderate-intensity activities (like fast walking, aerobic water sports, cycling on flat surfaces, and playing doubles tennis), and vigorous activities (such as running fast or mountain biking, basketball, professional swimming, and singles tennis), as per the Australian survey [12].

### Statistical methods

Based on the anthropometric data, the zBMI of the students was calculated using a WHO growth chart. The zBMI represents weight measurements adjusted for the children’s age and sex and is used for screening severe thinness, thinness, healthy weight, overweight, and obesity in children and adolescents [13]. Considering the different time points of data collection, the zBMI changes were used to represent the changes in weight status among the students.

After calculating the student’s zBMI along with the results of the qualitative variables, the results were presented using descriptive statistics. The collected demographic data and other information were analyzed with IBM SPSS Statistics for Windows version 24 software, and the statistical results are presented in the descriptive and analytical sections. All qualitative variables are presented as frequencies and percentages. Continuous quantitative variables are also presented as descriptive statistics (means and standard deviations). Due to the non-normal distribution of zBMI changes (determined using the Kolmogorov‒Smirnov test), we used nonparametric methods to analyze the significance of differences in zBMI changes among the studied variables (the Mann-Whitney U test for dichotomous variables and the Kruskal-Wallis H test for variables with more than two categories). The Wilcoxon signed-rank test was also used to compare the zBMI before and after the school closures. Comparisons between ordinal variables were evaluated using the Wilcoxon signed-rank test, and comparisons between nominal variables were evaluated using the McNamara test before and during school closures. In this study, *P* ≤ 0.05 was considered to indicate statistical significance.

## Results

The characteristics and details of the social-demographic data of the students are presented in Table 1. In this study, 100 students with an average age of 13.03 ± 1.2 years old were recruited for the final analysis, and over half of them (64%) were females. Among the parents of participants, 12% of mothers and 9% of fathers lost their jobs or worked from home and spent more time at home with their families. Moreover, the majority of the students (63%) lived in apartments with balconies, nearly half (45%) lived in families with two children, 38% lived in families with more than two children, and the rest lived in families with a single child.

**Table 1:** Demographic characteristics of the students in our study

Student’s Demographic data	Subgroups	Frequency (%)
Sex	Female	64(64%)
	Male	36(36%)
Age	10–12	11(11%)
	12–14	57(57%)
	14–16	32(32%)
Loss of mother’s job during lockdown	Yes	12(12%)
	No	88(88%)
Loss of father’s job during lockdown	Yes	9(9%)
	No	91(91%)
Residency Type	Apartment without balcony	11(11%)
	Apartment with balcony	63(63%)
	House without backyard	3(3%)
	House with backyard	23(23%)
Number of children in the family	Single child	17(17%)
	Two Children	45(45%)
	More than 2 children	38(38%)

According to Table 2, the zBMI increased significantly from − 0.02 ± 1.64 to 0.36 ± 1.12 (*P* ≤ 0.05) during the school closures. The BMI classification showed that thinness and severe thinness decreased from 8% pre-lockdown to 1% post-lockdown while healthy weight increased from 64% pre-lockdown to 68% post-lockdown. Additionally, there was a significant 3% rise in the overall population of individuals with overweight and obesity after the lockdown (*P* ≤ 0.05).

**Table 2:** Comparison of BMI before and after covid-19 lockdown

Variable	Pre-lockdown	Post-lockdown	*P*-Value
	Mean ± standard deviation	Mean ± standard deviation	
zBMI score	-0.02 ± 1.64	0.36 ± 1.12	0.03
**BMI Category**	**Frequency (%)**	**Frequency (%)**	
Thinness/ Severe thinness (z-score<-2SD))	8(8%)	1(1%)	0.04
normal weight (-2 < z-score < + 1SD)	64(64%)	68(68%)	
Overweight/Obesity (z-score > + 1SD)	28(28%)	31(31%)	

Table 3 presents changes in eating habits, including food responsiveness, food enjoyment, emotional overeating, and urgency to eat. Significant changes were observed in students, including a significant increase in food responsiveness and food enjoyment during quarantine (*P* ≤ 0.05). In the total population of students, a notable difference was observed in the tendency to overeat under the influence of stress and anxiety and the feeling of urgency to eat during quarantine (*P* ≤ 0.05). Moreover, regarding eating habits, the number of snacks consumed per day and week increased significantly. (*P* ≤ 0.05).

**Table 3:** Student’s eating habit status before and during covid-19 lockdown

Eating habits	Subgroup(*n* = 100)	Pre-lockdown frequency (%)	During lockdown frequency (%)	*P*-Value
I enjoy when I eat food	Strongly Disagree	5(5%)	8(8%)	< 0.001
	Disagree	6(6%)	5(5%)	
	Neutral	22(22%)	17(17%)	
	Agree	52(52%)	29(29%)	
	Strongly Agree	15(15%)	41(41%)	
I am a person with a good appetite	Strongly Disagree	7(7%)	10(10%)	< 0.001
	Disagree	12(12%)	7(7%)	
	Neutral	13(13%)	8(8%)	
	Agree	55(55%)	38(38%)	
	Strongly Agree	13(13%)	37(37%)	
Overeating under the influence of stress and mental anxiety	Never	60(60%)	58(58%)	< 0.001
	Sometimes	28(28%)	16(16%)	
	Often	9(9%)	20(20%)	
	Usually	2(2%)	2(2%)	
	Always	1(1%)	4(4%)	
The feel of urgency to eat	Never	63(63%)	54(54%)	< 0.001
	Sometimes	20(20%)	16(16%)	
	Often	15(15%)	25(25%)	
	Usually	2(2%)	4(4%)	
	Always	0(0%)	1(1%)	
Number of snacks consumed per week	Less than once a week	6(6%)	1(1%)	< 0.001
	1–3 times in a week	41(41%)	17(17%)	
	4–6 times in a week	31(31%)	31(31%)	
	Everyday	32(32%)	51(51%)	
Number of snacks consumed per day	Once a day	31(31%)	5(5%)	< 0.001
	Twice a day	45(45%)	26(26%)	
	3 times a day	23(23%)	42(42%)	
	4 or more than 4 times a day	1(1%)	27(27%)	

According to Table 4, the sleeping patterns of students before and during quarantine changed significantly (*p* < 0.001), and the dominant early-night sleep–early-morning wakeup rhythm (87%) before quarantine decreased from 87 to 50% during quarantine, while late-night sleep–late-morning wakeup rhythms increased from 13 to 50% in the sample population. These changes were significant during this period (*p* ≤ 0.05). Moreover, regarding hours of sleep, the data indicate a significant decrease (11%) in the number of students who had less than 8 h of sleep during lockdown. (*p* ≤ 0.05). In terms of sleeping quality, the periods of frequent awakenings during sleep in the study population increased significantly during quarantine (*p* ≤ 0.05), and there were no significant changes in frequent nightmares during sleep or difficulty falling asleep (*p* > 0.05).

**Table 4:** Student’s sleeping status before and during covid-19 lockdown

Sleeping status	Subgroup(*n* = 100)	Pre-lockdown frequency (%)	During lockdown frequency (%)	*P*-Value
Sleeping Pattern	Early-night sleep–early-morning wakeup rhythm	87(87%)	50(50%)	< 0.001
	Late-night sleep–late-morning wakeup rhythm	13(13%)	50(50%)	< 0.001
Hours of Sleep	Less than 8 h	26(26%)	15(15%)	< 0.001
	8–10 h	57(57%)	57(57%)	
	More than 10 h	17(17%)	28(28%)	
Sleeping Quality	Frequent waking periods during sleep	12(12%)	24(24%)	< 0.001
	Frequent nightmares during sleep	15(15%)	22(22%)	0.21
	Difficulty falling asleep	30(30%)	27(27%)	0.54

Table 5 indicates the amount of screen time and frequency of the students engaged in physical activities. The percentage of students with low-, moderate-, and vigorous-intensity physical activity decreased from 87% before quarantine to 52% during quarantine, and these changes were significant during this period (*p* ≤ 0.05). Additionally, the number of students engaged in sedentary activities (almost all sitting activities) rose significantly from 13 to 48% during quarantine (*p* ≤ 0.05). The results also indicate a significant rise in the use of social media during quarantine, and the majority of students (71%) spent more than 3 h per day watching television and using computer games and mobile phones (*P* ≤ 0.05).

**Table 5:** Student’s physical activity and screen time status before and during the covid-19 lockdown

Physical activity & screen timing	Subgroup(*N* = 100)	Pre-lockdown frequency (%)	During lockdown frequency (%)	*P*-Value
Physical Activity	Sedentary activity (almost all sitting-based activities)	13(13%)	48(48%)	< 0.001
	Light-intensity activity (include domestic or occupational tasks such as washing dishes, hanging washing, ironing, cooking, eating, working at a computer desk, or performing other office duties)	19(19%)	37(37%)	
	Moderate intensity (including fast walking, aerobic water sports, cycling on flat surfaces, and playing doubles tennis)	56(56%)	15(15%)	
	Vigorous-intensity activity (running, fast or mountain biking, basketball, professional swimming, and singles tennis)	12(12%)	0(0%)	
Screen timing	0–1 h	22(22%)	3(3%)	< 0.001
	1–2 h	41(41%)	8(8%)	
	2–3 h	19(19%)	18(18%)	
	more than 3 h	18(18%)	71(71%)	

Table 6 illustrates the changes in zBMI among the studied predictor factors during school closures. According to the table, zBMI changes were significantly greater for males and students aged 14–16 years old (*P* ≤ 0.05). The increase in zBMI was also notably greater in children who reported an increase in food enjoyment, food responsiveness, and late-night sleep–late morning-wakeup rhythms, as well as a decrease in physical activity during quarantine (*p* ≤ 0.05).

**Table 6:** zBMI changes in studied variables

Variable	Subgroups	zBMI change mean ± SD	*p*-value
Sex	Female	0.10 ± 0.85	**< 0.001**
	Male	0.90 ± 1.61	
Age	10–12	1.12 ± 1.62	**0.01**
	12–14	0.19 ± 1.07	
	> 14	0.49 ± 1.29	
Loss of mother’s job during lockdown	Yes	0.67 ± 1.07	0.19
	No	0.36 ± 1.25	
Loss of father’s job during lockdown	Yes	-0.05 ± 0.35	0.19
	No	0.45 ± 1.30	
Residency Type	Apartment without balcony	0.68 ± 1.36	0.50
	Apartment with balcony	0.30 ± 1.18	
	House without backyard	0.72 ± 0.65	
	House with backyard	0.44 ± 1.40	
Number of children in the family	Single child	0.22 ± 0.75	0.32
	Two Children	0.68 ± 1.61	
	More than 2 children	0.12 ± 0.75	
Change in emotional overeating	Decrease	-0.05 ± 0.50	0.12
	No Change	0.39 ± 1.36	
	Increase	0.62 ± 1.09	
Change in urgency to eat	Decrease	0.13 ± 0.38	0.11
	No Change	0.29 ± 1.24	
	Increase	0.82 ± 1.20	
Change in the enjoyment of food	Decrease	-0.24 ± 0.32	**< 0.001**
	No Change	0.30 ± 1.23	
	Increase	0.81 ± 1.42	
Change in food responsiveness	Decrease	-0.10 ± 0.52	**< 0.001**
	No Change	0.19 ± 1.24	
	Increase	1.02 ± 1.46	
Change in the frequency of snack consumption per week	Decrease	0.07 ± 0.00	0.71
	No Change	0.24 ± 1.13	
	Increase	0.51 ± 1.32	
Change in the frequency of snack consumption per day	Decrease	-	0.16
	No Change	0.26 ± 1.37	
	Increase	0.43 ± 1.19	
Change in sleeping time	Decrease	0.21 ± 1.09	0.99
	No Change	0.36 ± 1.50	
	Increase	0.18 ± 0.90	
Change in sleeping patterns	With Change	0.66 ± 1.33	**0.01**
	Without Change	0.12 ± 1.08	
Change in the quality of sleep (periods of frequent awakening during sleep)	With Change	0.39 ± 0.72	0.31
	Without Change	0.38 ± 1.32	
Change in sleeping quality (frequent nightmares during sleep)	With Change	0.27 ± 0.90	0.99
	Without Change	0.42 ± 1.32	
Change in sleeping quality (difficulty falling asleep)	With Change	0.03 ± 0.87	0.41
	Without Change	0.43 ± 1.27	
Change in screen time	Decrease	0.46 ± 0.94	0.51
	No Change	0.02 ± 0.78	
	Increase	0.47 ± 1.33	
Change in Physical Activity	Decrease	0.63 ± 1.32	**< 0.001**
	No Change	-0.20 ± 0.72	
	Increase	-	

## Discussion

In this descriptive cross-sectional study, we investigated changes in zBMI, eating habits, and lifestyle among students aged 10–16 years old following school closures during the COVID-19 lockdown. Our study took place at the end of quarantine and after schools reopened in April-May 2022. The findings revealed significant shifts in weight gain, negative eating behaviors, increased social media usage, altered sleep patterns, and reduced physical activity among students during the quarantine period.

recent studies have found an increase in the prevalence of overweight and obesity among children and adolescents, ranging from 7 to 13% during quarantine [9, 14–17], suggesting higher rates compared to our study (3%). These differences may be due to variations in the timeframes for measuring and comparing participants’ weight and height, as well as differences in sample sizes. Moreover, previous studies confirmed that children and adolescents experienced weight gain during quarantine, which aligns with our research findings; however, these studies relied more on self-reported data obtained through online or offline surveys regarding weight changes during quarantine, without accurately knowing the exact weight and height of the participants before quarantine, which could introduce recall bias and affect the accuracy of the collected information [1, 3, 6, 9, 18]. On the other hand, our study obtained students’ weight and height data from records maintained by the school principals or physical education instructors before and after the quarantine period. This approach aimed to provide more precise and reliable information, reducing the likelihood of recall bias and ensuring a more accurate representation of the students’ changes in weight and height. We also used the BMI-for-age z-score of the students to compare their weight with that of others of the same age and sex. This approach is consistent with the study conducted in Qatar, which used the same method to compare weight changes among children during the lockdown. Consistent with our study, they conducted a long-term analysis and used electronic health records of children’s weight and height to compare children’s age- and sex-adjusted BMI before and after the COVID-19 restrictions, which significantly increased (0.30) during the lockdown [15].

In agreement with our findings, two previous studies reported that males experienced greater increases in zBMI changes than females during quarantine [15, 19]. Additionally, we realized that there was a significant increase in zBMI among 14-16-year-old students, while Abed Alah et al. reported the greatest increase in zBMI among younger children aged 8–11 years [15]. This discrepancy may be linked to the larger sample size, which included 1546 students, and the different age categories that were divided into individuals aged 8–11 and 12–15 years old, in their study.

Regarding the impact of the number of children in the family on students’ weight changes, our findings differed from those of Ghandi et al., which indicated a notable correlation between the number of children and childhood obesity [20]. One interpretation of these differences could be that parents had the opportunity to closely monitor their children’s diet during quarantine as they spent more time at home together.

During the quarantine period, eating habits were significantly influenced by factors like reduced food access, limited grocery hours, and increased consumption of unhealthy foods [21]. Our findings revealed that students experienced increased levels of food responsiveness, food enjoyment, emotional overeating, and urgency to eat followed by the quarantine and school closures. In addition, we observed an increase in the frequency of snack consumption by students during the day and the week. Increased child boredom is one of the factors that can explain increased levels of food responsiveness, emotional consumption overeating, and snack frequency during lockdown [10]. In a study by Morres et al., similar patterns were observed, consistent with our findings. They highlighted deteriorating eating behavior among adolescents aged 12 to 17, including an increase in the number of main meals and snacks. This study also mentioned a decline in dietary restraint, reduced meal quality, and an increase in the consumption of sugary food items [6]. Another study conducted in Egypt in 2020 also noted changes in eating habits among the majority of children (73.5%) and reported an increase in the number of their consumed meals (3–4 servings per day). These findings are directly in line with the results observed in our study [22].

Our study also highlighted a significant change in students’ zBMI based on alterations in their eating habits. Consistent with our findings, an Italian study in 2020 reported that BMI was positively correlated with increased appetite and night snacks [23]. Moreover, a study by Umano et al. reported that the rate of increased food responsiveness during the COVID-19 lockdown was greater in normal-weight children than in those with obesity, which was in line with our study [24].

Regarding the students’ sleeping status during lockdown, Androutsos et al.‘s study on changes in sleeping patterns among children and adolescents before and during quarantine revealed an increase of 10.9% in the number of individuals sleeping more than 10 h compared to pre-lockdown hours which aligned with our results [1]. Supporting our study’s outcomes, Ranjbar et al. corroborated the increase in sleeping duration among students during quarantine. Their study attributed this increase in sleeping hours to a greater amount of time spent using social media and a decreased inclination among students toward engaging in artistic and physical activities during the quarantine period [7]. Contrary to the findings of Androutso et al., who reported a significant link between increased sleeping duration and changes in the weight of children and adolescents, we did not observe a significant rise in zBMI in children who had more sleeping hours [1]. One plausible explanation for these discrepancies could be the small sample size in our study. Additionally, our study compared behavioral sleeping patterns over a longer period (two years), potentially leading to a greater likelihood of recall bias or modified changes in weight gain due to increased sleeping time compared to the shorter time intervals investigated in the aforementioned studies (less than one year).

Increased sleeping problems in our study support the findings of the study conducted by Pisano et al., which indicated sleeping problems in children during quarantine, reporting that 19.99% experienced issues such as difficulty falling asleep, frequent awakenings, and agitation during sleep [11]. An increase in sleeping disorders and changes in sleeping duration pose a risk factor for weight gain and contribute to the prevalence of overweight and obesity during quarantine [25]. Our study revealed a significant increase in zBMI among students who experienced alterations in their sleeping patterns, particularly those who shifted from an early-night sleep–early-morning wakeup rhythm to a late-night sleep–late-morning wakeup rhythm. Changes in students’ sleeping time and quality may be influenced by increased use of social media and exposure to blue light emitted by mobile phones, tablets, and monitors [26]. Ranjbar et al. reported a direct correlation between augmented social media usage and extended sleeping hours among students [7].

Our research also revealed a significant rise in the duration of social media use during quarantine. The surge in social media usage can be attributed to the prolonged period of virtual education that replaced in-person classes during quarantine. Additionally, increased leisure time at home and reduced physical activity among children and adolescents have contributed to this trend. Several studies reached similar conclusions, confirming the increased screen time during the lockdown [1, 3, 18, 27, 28]. In contrast to our findings, which showed no significant increase in zBMI among students with extended screen time, Androutsos et al.‘s study revealed a significant correlation between screen time and body weight increase in children and adolescents [1].

Related to the student’s physical activity, some studies echoed our findings, emphasizing a decrease in physical activity and an increase in sedentary time among children and adolescents during quarantine [1, 3, 5, 18, 29]. This reduction in physical activity can exacerbate mental health issues and have adverse effects on overall well-being [7].

Our study revealed a significant increase in the zBMI of students with decreased physical activity during school closures. Consequently, a decrease in physical activity can serve as a predictive factor contributing to the increasing prevalence of obesity and overweight among children and adolescents. Previous studies also supported this connection between reduced physical activity and weight gain in children during quarantine, consistent with our research findings [1, 15].

This study has several limitations. The first was related to its cross-sectional design. Second, the sample size may not reflect the situation of all students; therefore, further studies with larger sample sizes are recommended. The third important limitation of this study is recall bias. Since the relevant questionnaire examined the lifestyle changes of the students before and during the quarantine, and this survey was conducted at a two-year interval; there is a possibility of forgetting and incomplete recall of the information. This limitation does not apply to the weight and height reports of the students because the information on the weight and height of the students was obtained from the data recorded by the physical education instructors or the school principal before the quarantine. Lastly, the focus of this paper was not on presenting the regression model. However, based on available data, we plan to delve deeper into various regression models in future research within this field.

## Conclusion

In this study, we observed a significant increase in BMI-for-age z-score during the quarantine period among children and adolescents. This increase was more pronounced among males and students aged 14–16 years old. Furthermore, we found significant negative changes in eating habits, sleeping time, sleeping patterns, screen time, and physical activity during quarantine. Specifically, students who experienced negative eating behaviors, altered sleeping patterns, and decreased physical activity during school closures showed a significant rise in zBMI.

Regarding these negative changes, it is crucial for the government, schools, health professionals, and parents to recognize the substantial impact of COVID-19 quarantine on the lifestyles of children and adolescents. Establishing an appropriate framework for students’ diet, physical activity, and sleeping quality is essential to facilitate weight control and reduce the prevalence of obesity and overweight.

Additionally, it is vital to consider default plans in the event of a resurgence of widespread contagion and subsequent school closures, or during school summer vacations when students are deprived of the structured nutritional and exercise programs provided by schools. These plans can help prevent the adverse effects resulting from the absence of such programs.

### Electronic supplementary material

Below is the link to the electronic supplementary material.

Supplementary Material 1

## Data Availability

No datasets were generated or analysed during the current study.

## Abbreviations

SARS-CoV-2: Severe acute respiratory syndrome coronavirus 2
WHO: World Health Organization
zBMI: BMI z-score
