# Supplementary material for: Long-term effects of the COVID-19 lockdown on weight status, eating habits, and lifestyle changes related to school-aged children in Bandar Abbas, Iran
Source: BMC Public Health. 2024 Jul 24;24:1981. doi: 10.1186/s12889-024-19509-3 (PMC11270933; doi:10.1186/s12889-024-19509-3)
Supplement: Supplementary file 1 — Supplementary Material 1 [file 12889_2024_19509_MOESM1_ESM.pdf]

Dear student! This survey has been created to assess your physical and mental well-being throughout the 2 years of COVID lockdown and school closures. If you are interested in participating in this research, kindly respond to the following questions.

We would like to emphasize that all relevant data in the questionnaire will be kept confidential and the results will be interpreted for participants if they choose to do so. Writing the name and surname is not a requirement

**Demographic data**	
**gender**<br>female□<br>male□	**Date of birth:**
**mother's job status during lockdown Loss of**<br>yes □<br>no □	**father's job status during lockdown Loss of**<br>yes □<br>no □
**Residency Type**<br>Apartment without balcony□<br>Apartment with balcony□<br>A house without a backyard□<br>A house with a backyard □	**Number of children in the family**<br>Single child □ two children □<br>more than two children□

**Dietary habits**						
		**Never**	**Sometimes**	**Often**	**Usually**	**Always**
**Overeating under the influence of stress and mental anxiety**	**Before lockdown**					
	**During lockdown**					
**The feeling of urgency to eat**	**Before lockdown**					
	**During lockdown**					
		**Strongly Disagree**	**Disagree**	**Neutral**	**Agree**	**Strongly Agree**
**I enjoy when I eat food**	**Before lockdown**					
	**During lockdown**					
**I am a person with a good appetite**	**Before lockdown**					
	**During lockdown**					

Number of snacks per week				
	Less than once a week	1-3 times in a week	4-6 times in a week	Everyday
Before lockdown				
During lockdown				
**Number of snacks consumed per day**				
	Once a day	Twice a day	3 times a day	4 or more than 4 times a day
Before lockdown				
During lockdown				

Sleep Pattern									
Before lockdown	Night-Sleep/Day-Awake			Day-Sleep/Night-Awake					
During lockdown	Night-Sleep/Day-Awake			Day-Sleep/Night-Awake					
**Hours of Sleep**									
Before lockdown	Less than 8 hours			8-10 hours			More than 10 hours		
During lockdown	Less than 8 hours			8-10 hours			More than 10 hours		
**Sleep Quality**									
		Yes (More than 2-3 times a week)	No (Less than 2-3 times a week)		Yes (More than 2-3 times a week)	No (Less than 2-3 times a week)		Yes (More than 2-3 times a week)	No (Less than 2-3 times a week)
Before lockdown	Frequent waking periods during sleep			Frequent nightmares during sleep			Difficulty falling asleep		
During lockdown	Frequent waking periods during sleep			Frequent nightmares during sleep			Difficulty falling asleep		

Screen time (daily amount of time spent using a device with a screen such as a smartphone, computer, television, video game, console, or even tablet.)								
**Before lockdown**	**0-1 hours**		**1-2 hours**		**2-3 hours**		**more than 3 hours**	
**During lockdown**	**0-1 hours**		**1-2 hours**		**2-3 hours**		**more than 3 hours**	

Physical Activity		
**Before lockdown**	**Just Sedentary activity (almost all sitting-based activity)**	
	**Light-intensity activity (include domestic or occupational tasks such as washing dishes, hanging washing, ironing, cooking, eating, and working at a computer desk)**	
	**Moderate intensity (including fast walking, aerobic water sports, cycling on flat surfaces, and playing doubles tennis)**	
	**Vigorous-intensity activity (running, fast or mountain biking, basketball, professional swimming, and singles tennis)**	
**During lockdown**	**Just Sedentary activity (almost all sitting-based activity)**	
	**Light-intensity activity (include domestic or occupational tasks such as washing dishes, hanging washing, ironing, cooking, eating, and working at a computer desk)**	
	**Moderate intensity (including fast walking, aerobic water sports, cycling on flat surfaces, and playing doubles tennis)**	
	**Vigorous-intensity activity (running, fast or mountain biking, basketball, professional swimming, and singles tennis)**	
